# Association between physical fitness and myopia among school-entry students: a school-based cross-sectional study

**DOI:** 10.3389/fpubh.2026.1916100

**Published:** 2026-09-03

**Authors:** ChengChao Duan, Yi Ba, Honglin Song, Yaqi Xue

**Affiliations:** 1 College of P.E. and Sports, Beijing Normal University, Beijing, China; 2 Division of Sports Science and Physical Education, Tsinghua University, Beijing, China

**Keywords:** motor fitness, myopia, physical fitness, school health surveillance, SHAP, students, XGBoost

## Abstract

**Objective:**

We examined whether school physical-fitness performance was associated with screening-defined current myopia in primary grade 1 and junior middle grade 1 students. We also explored item-level fitness profiles in relation to current myopia status.

**Methods:**

We analyzed routine health-screening records collected in 2025 from three schools in Xicheng District, Beijing. Screening-defined current myopia required unaided visual acuity <5.0 together with a non-cycloplegic spherical equivalent ≤ −0.50 D in the same eye. Official fitness scores were recalculated under the National Student Physical Health Standard (2014 revision). Prevalence ratios (PRs) were estimated with modified Poisson regression, and continuous associations were assessed using restricted cubic splines. Sensitivity analyzes incorporated school fixed effects and class-clustered standard errors. XGBoost and SHAP were used only to explore patterns in the observed data.

**Results:**

The analysis included 1,296 students: 642 in primary grade 1 and 654 in junior middle grade 1. Current myopia by the screening definition was identified in 496 students (38.3%), with prevalence of 6.7 and 69.3% in the two grade-stage groups, respectively. In the original adjusted models, students who failed the fitness standard had a higher myopia prevalence than those rated excellent (PR = 1.95, 95% CI: 1.36–2.79). A 1-SD lower motor-fitness score was also associated with higher prevalence (PR = 1.14, 95% CI: 1.03–1.26). After accounting for school and within-class clustering, the fail-versus-excellent estimate remained evident (PR = 1.95, 95% CI: 1.13–3.38); so did the estimate for lower motor fitness in primary grade 1 (PR = 2.05, 95% CI: 1.13–3.69). The remaining estimates pointed in similar directions but were less precise. Internal machine-learning performance was moderate, and item attributions varied by model and grade stage.

**Conclusion:**

Failing the official fitness standard and having lower motor fitness were associated with screening-defined current myopia in this cross-sectional sample. The clearest association was found in primary grade 1. These results cannot establish which condition came first and may reflect non-cycloplegic misclassification, reverse causation, or shared behavioral, familial, developmental, educational, and environmental influences. Fitness measures are not ready for use in myopia prediction or screening.

## Introduction

Myopia is now common among children and adolescents. Its prevalence has risen markedly in recent decades and is projected to continue increasing through 2050 (1, 2). Earlier onset matters: it allows more time for progression and raises the lifetime risk of high myopia and related ocular complications (3, 4). School vision screening offers a practical route to early identification and referral, while also supporting health education and population surveillance.

A surveillance definition must work in the field. It should be reproducible, feasible at scale, and clear about what it does and does not diagnose. Pairing unaided visual acuity with an eye-specific spherical equivalent draws on both functional vision and refractive information in routine records (5, 6). Such a definition is useful for school monitoring and referral, although it does not replace a cycloplegic examination.

Daily life at school brings together several established or suspected correlates of myopia. Additional outdoor time has reduced myopia incidence in school-based randomized research, and meta-analyzes support a preventive role for outdoor exposure (7–10). Near work and digital-device use have also been linked to myopia, although both overlap with educational demands and sedentary time (11–13). Recent work further suggests a dose–response relation with screen time and places physical activity, sedentary behavior, and sleep within the same behavioral setting (14, 15).

School fitness testing captures a different, but partly overlapping, view of student health. Under the National Student Physical Health Standard, the total score combines body morphology, cardiopulmonary function, speed, flexibility, coordination, strength, and endurance (16). It is not a single biological measure. Performance can reflect activity and outdoor participation, but also growth, maturation, sedentary habits, and the routines of a particular school stage.

Primary grade 1 and junior middle grade 1 mark two distinct school-entry periods. Students differ in age, accumulated educational exposure, academic demands, test items, and daily schedules. Education is closely linked to myopia, and schools are central to prevention efforts (17, 18). Yet a comparison of two grade stages at one time point cannot show incidence, progression, or change within a child. We therefore examined cross-sectional associations between physical-fitness performance and current myopia at these two entry stages, with an additional exploratory analysis of item-level fitness profiles.

## Materials and methods

### Study design and participants

We conducted a school-based cross-sectional study during the 2025 routine health-screening program in Xicheng District, Beijing, China, and followed STROBE recommendations for observational research (27). Administrative school zones formed the sampling strata. Eligible schools were listed from administrative records, and a computer-generated random-number procedure selected three schools from different zones. Every primary grade 1 and junior middle grade 1 student in those schools was invited. The frame was deliberately local: it was not designed to represent Beijing or China as a whole. Because students were nested within classes and schools, shared educational, environmental, and testing practices could make observations within a cluster more alike.

Eligibility required complete routine records for vision screening, non-cycloplegic refraction, anthropometry, and official fitness testing. We reviewed the ocular-history fields before constructing the analytic dataset. Records indicating amblyopia, congenital cataract, corneal disease, retinal disease, or previous ocular surgery were excluded. Other exclusions were absence from vision or fitness testing; missing outcome, exposure, or covariate data; implausible values identified during data-quality review; and a recorded medical condition that precluded standard fitness testing. The final sample comprised 1,296 students.

### Definition of myopia

The outcome was current myopia as defined by the school screening protocol. All three schools followed WS/T 663-2020, the national Specification for Screening of Refractive Error in Primary and Secondary School Students. Trained health personnel measured unaided distance visual acuity in each eye at 5 m using a standard logarithmic chart conforming to GB/T 11533-2011. The chart surface received at least 300 lx of illumination (or the white background had luminance of at least 200 cd/m^2^); lighting was uniform and stable, without glare or reflection. The fellow eye was covered without pressure. Non-cycloplegic refraction was obtained with a Topcon KR-800 automated refractometer (Topcon Corporation, Tokyo, Japan). The same operating procedures were used in each school. At the start of each screening day, staff checked the instrument with a standard model eye and set cylinder power to negative notation. Three readings were taken per eye and averaged to two decimal places. If any two spherical-power readings differed by ≥0.50 D, staff repeated the measurement and recalculated the mean. Spherical equivalent (SE) equaled sphere plus one-half cylinder. A student met the screening definition when unaided visual acuity was <5.0 and non-cycloplegic SE was ≤−0.50 D in the same eye, in either eye. This was a surveillance outcome, not a cycloplegic clinical diagnosis.

### Physical fitness and anthropometric measures

Height and weight came from routine school physical-examination records; BMI was calculated as weight in kilograms divided by height in meters squared. Fitness testing followed the National Student Physical Health Standard (2014 revision). We recalculated the official total score from the recorded items. Supplementary Table S1 gives the grade- and sex-specific conversion rules, item weights, bonus rules, and final-score calculation.

The available indicators were total score, fitness level, vital capacity, 50-m run, sit-and-reach, rope skipping, standing long jump, endurance run, sit-ups, and pull-ups. Raw motor items were standardized within grade-stage strata. We reversed the coding of timed runs so that a higher standardized value always denoted better performance, then averaged the available standardized motor items to form the motor-fitness indicator. Vital capacity was kept separate because of its strong dependence on growth, maturation, and body size. In the domain analysis, standardized vital capacity represented functional fitness and was analyzed apart from motor fitness.

### Statistical analysis

We summarized continuous variables as means and standard deviations and categorical variables as counts and percentages. Because myopia was common, we estimated PRs and 95% confidence intervals (CIs) using modified Poisson regression with robust standard errors (19, 20). Main models included grade-stage stratum, sex, and BMI; models within a grade stage included sex and BMI. Total score was expressed per 10-point decrease, with excellent fitness as the reference category for fitness level. We then repeated the models with school fixed effects and standard errors clustered by class. The data contained three schools and 36 classes. Student t inference used 35 degrees of freedom for the full sample and 17 degrees of freedom within each grade-stage stratum. We did not use conventional school-clustered inference because three school clusters are too few for reliable variance estimation. OpenAI Codex was used solely for English-language polishing and was not used to implement, perform, or check the statistical or sensitivity analyses.

Restricted cubic splines were fitted to the continuous fitness total score. The median score served as the reference, and models adjusted for grade-stage stratum, sex, and BMI. We report tests for the overall association and for non-linearity (21, 22).

### Machine-learning analysis of physical-fitness profiles

We fitted XGBoost models separately in primary grade 1 and junior middle grade 1 to examine whether item-level fitness profiles distinguished students who met the screening definition from those who did not (23, 24). Each stratum had three model sets: baseline (sex, height *z*-score, and body-weight *z*-score), fitness items alone, and baseline plus fitness items. Performance was evaluated by repeated stratified 5-fold cross-validation. AUC measured discrimination, Brier score measured probability error (25), and SHAP values summarized feature attributions within each fitted model (26). This was an exploratory analysis. It was neither designed nor validated as a clinical screening tool.

## Results

### Participant characteristics and grade-stage burden

The analytic sample comprised 1,296 students: 642 in primary grade 1 and 654 in junior middle grade 1. There were 719 boys and 577 girls. Overall, 496 students (38.3%) met the screening definition for current myopia. The contrast by grade stage was marked: 43 of 642 primary grade 1 students (6.7%) versus 453 of 654 junior middle grade 1 students (69.3%). Mean SE was +0.08 D in the younger group and −1.76 D in the older group. These figures compare different groups at one time point; they do not describe progression within the same students.

The sex pattern also differed between grade stages. In primary grade 1, prevalence was 7.6% in boys and 5.3% in girls. The corresponding values in junior middle grade 1 were 65.4 and 73.0%. After adjustment, girls in junior middle grade 1 had a PR of 1.11 relative to boys (95% CI: 1.00–1.23; Tables 1, 2).

**Table 1:** Baseline characteristics by grade-stage stratum.

Characteristic	Primary grade 1	Junior middle grade 1	Total
*n*	642	654	1,296
Male, *n* (%)	395 (61.5)	324 (49.5)	719 (55.5)
Female, *n* (%)	247 (38.5)	330 (50.5)	577 (44.5)
BMI, mean (SD)	15.35 (1.99)	19.39 (3.22)	17.39 (3.36)
Mean SE, D, mean (SD)	0.08 (0.94)	−1.76 (1.77)	−0.85 (1.69)
Myopia, *n* (%)	43 (6.7)	453 (69.3)	496 (38.3)
Official fitness total score, mean (SD)	88.74 (12.00)	83.03 (10.26)	85.86 (11.51)

**Table 2:** Myopia by grade-stage and sex.

Stratum	Boys	Girls
Primary grade 1	30/395 (7.6%)	13/247 (5.3%)
Junior middle grade 1	212/324 (65.4%)	241/330 (73.0%)
Total	242/719 (33.7%)	254/577 (44.0%)

### Physical fitness and myopia

Myopia prevalence rose as the official fitness category declined, although estimates for the intermediate categories were close to the reference. Relative to excellent fitness, adjusted PRs were 1.09 (95% CI: 0.96–1.25) for good, 1.04 (95% CI: 0.88–1.22) for pass, and 1.95 (95% CI: 1.36–2.79) for fail. For the continuous score, the overall estimate was smaller: PR = 1.05 per 10-point decrease (95% CI: 0.99–1.12).

The motor-fitness result was more pronounced. Each 1-SD decrease in the motor-fitness *z*-score corresponded to a PR of 1.14 (95% CI: 1.03–1.26). This pattern came mainly from primary grade 1, where a 10-point lower total score had a PR of 1.39 (95% CI: 1.03–1.88) and a 1-SD lower motor-fitness *z*-score had a PR of 2.12 (95% CI: 1.40–3.22). We did not observe comparable associations in junior middle grade 1.

**Figure 1:**
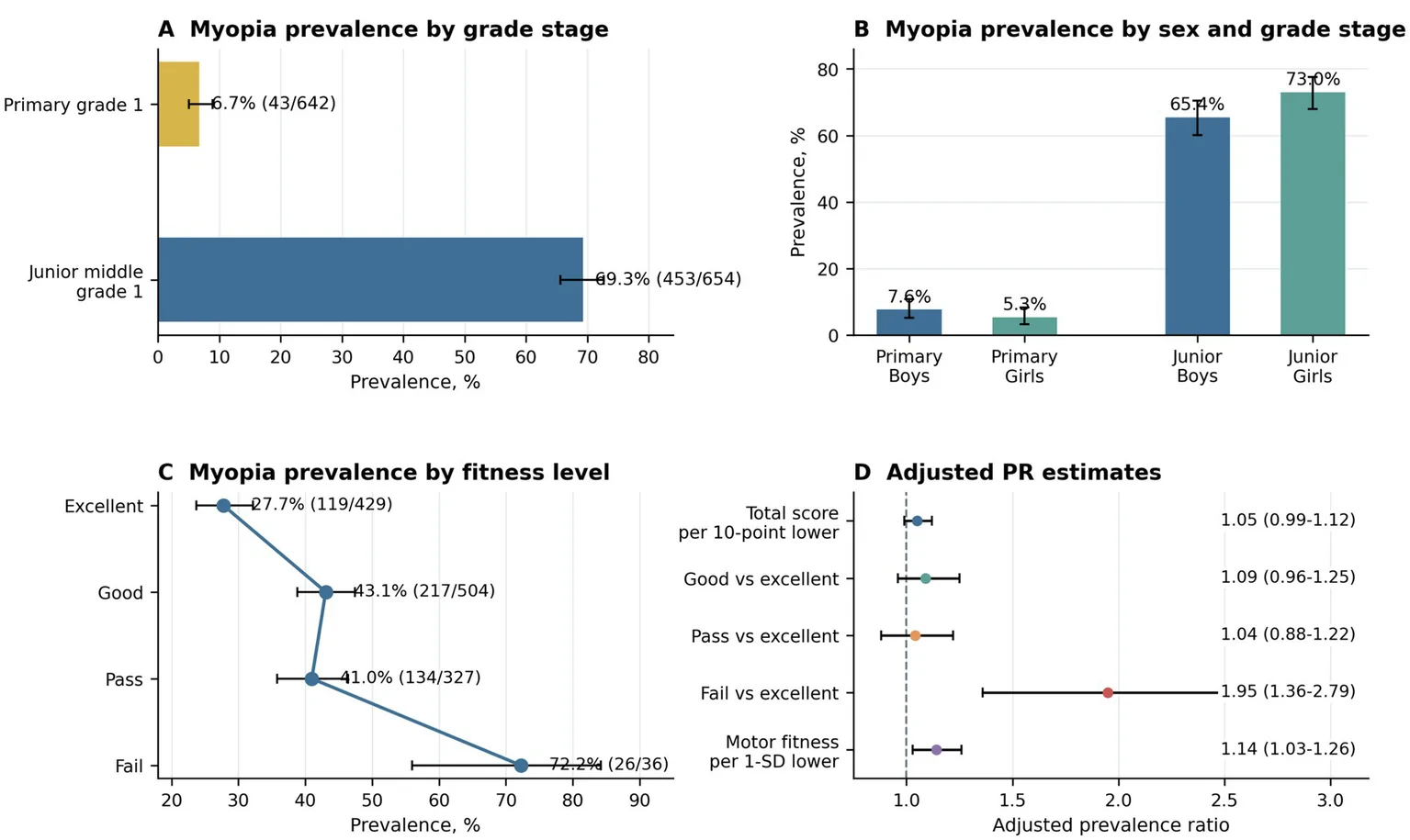
Grade-stage burden and physical-fitness associations with myopia. **(A)** Prevalence by grade-stage stratum. **(B)** Prevalence by sex within grade-stage stratum. **(C)** Prevalence across official fitness levels. **(D)** Adjusted PR estimates for total score, fitness level, and motor fitness.

**Figure 2:**
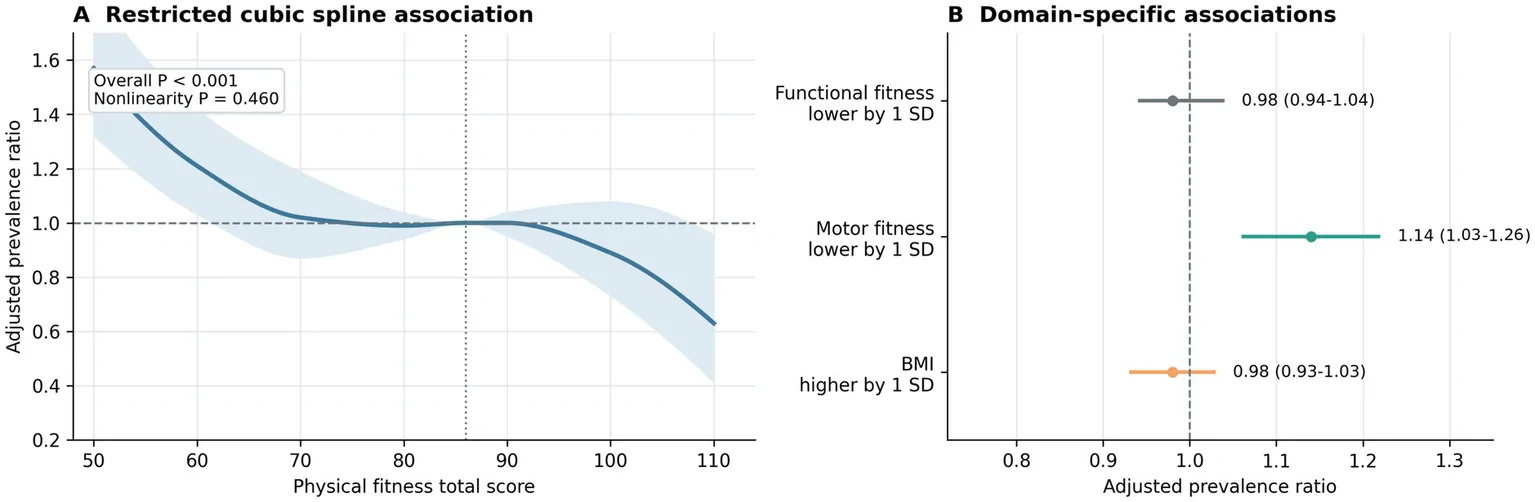
Continuous and domain-specific associations of physical-fitness indicators. **(A)** The restricted cubic spline curve shows the adjusted prevalence ratio across the physical-fitness total score range. **(B)** The domain-specific forest plot compares functional fitness, motor fitness, and BMI indicators.

Accounting for school and within-class clustering widened several confidence intervals. The fail-versus-excellent estimate remained PR = 1.95 (95% CI: 1.13–3.38; *p* = 0.018), and the primary-grade estimate for lower motor fitness was PR = 2.05 (95% CI: 1.13–3.69; *p* = 0.020). Estimates for overall motor fitness (PR = 1.14, 95% CI: 0.99–1.31; *p* = 0.078) and primary-grade total score (PR = 1.37, 95% CI: 0.88–2.13; *p* = 0.150) were less precise. Supplementary Table S2 provides the full set of results (Table 3; Figures 1, 2).

**Table 3:** Association of physical fitness indicators with myopia.

Indicator	*n*/events	Adjusted PR (95% CI)	*p* value
Fitness total score, per 10-point lower	1,296/496	1.05 (0.99–1.12)	0.100
Fitness level: excellent	429/119	1.00 (reference)	–
Fitness level: good	504/217	1.09 (0.96–1.25)	0.190
Fitness level: pass	327/134	1.04 (0.88–1.22)	0.640
Fitness level: fail	36/26	1.95 (1.36–2.79)	<0.001
Motor fitness, per 1-SD lower	1,296/496	1.14 (1.03–1.26)	0.014
Primary grade 1: total score, per 10-point lower	642/43	1.39 (1.03–1.88)	0.031
Primary grade 1: motor fitness, per 1-SD lower	642/43	2.12 (1.40–3.22)	<0.001
Junior middle grade 1: total score, per 10-point lower	654/453	1.01 (0.96–1.06)	0.758
Junior middle grade 1: motor fitness, per 1-SD lower	654/453	1.03 (0.94–1.12)	0.529

### Physical-fitness profiles and current myopia status

Within this dataset, item-level fitness profiles contained some information about current myopia status. In primary grade 1, the fitness-items model had a higher cross-validated AUC than the anthropometric baseline model (0.824 vs. 0.746), accompanied by a lower Brier score. Adding fitness items in junior middle grade 1 increased AUC from 0.648 to 0.721 and also reduced the Brier score. These are internal estimates. They do not demonstrate clinical usefulness, and their performance in a new population is unknown.

**Figure 3:**
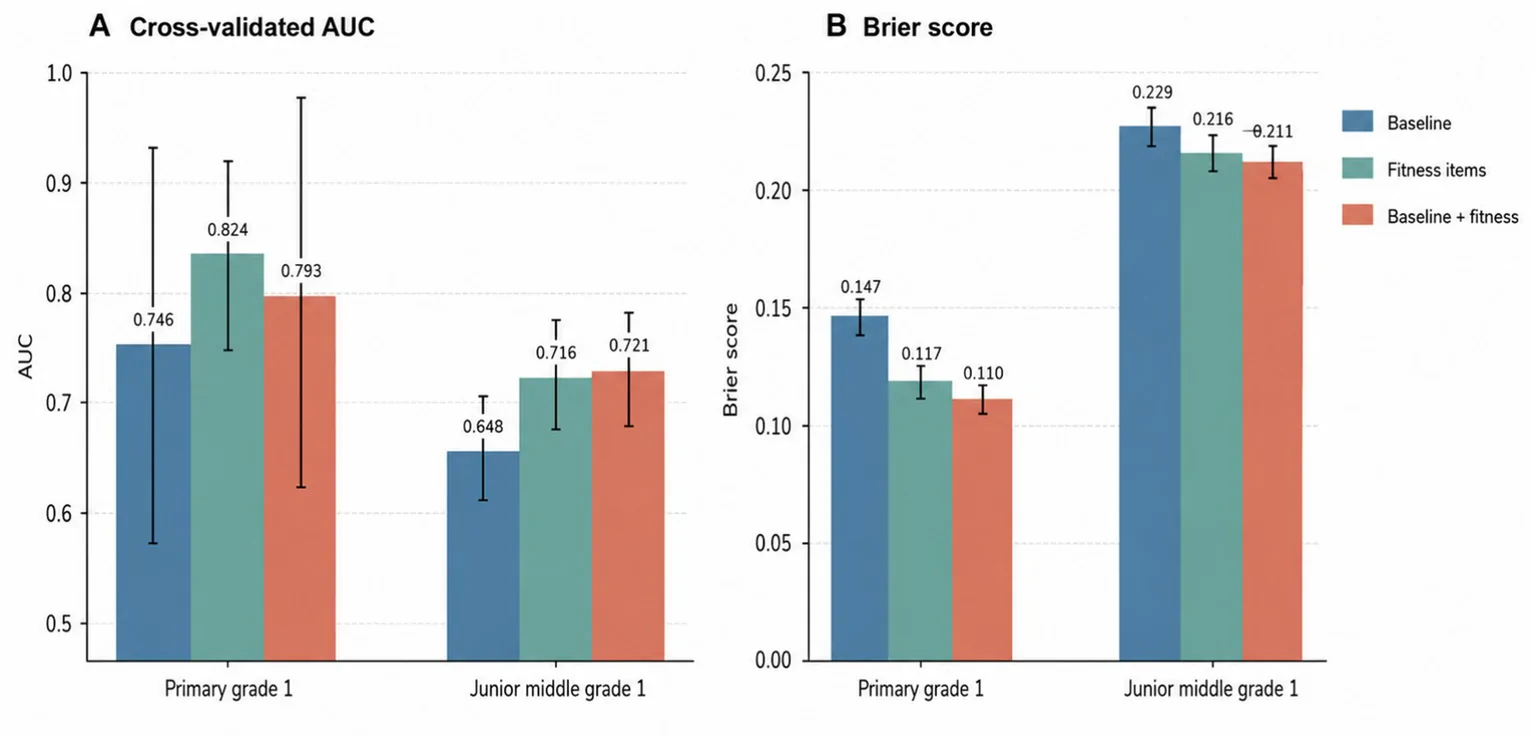
Machine-learning model performance for current myopia status. Models were fitted separately within each grade-stage stratum using baseline anthropometric variables, item-level fitness indicators, or baseline + fitness variables. Bars show mean cross-validated AUC and Brier score.

**Figure 4:**
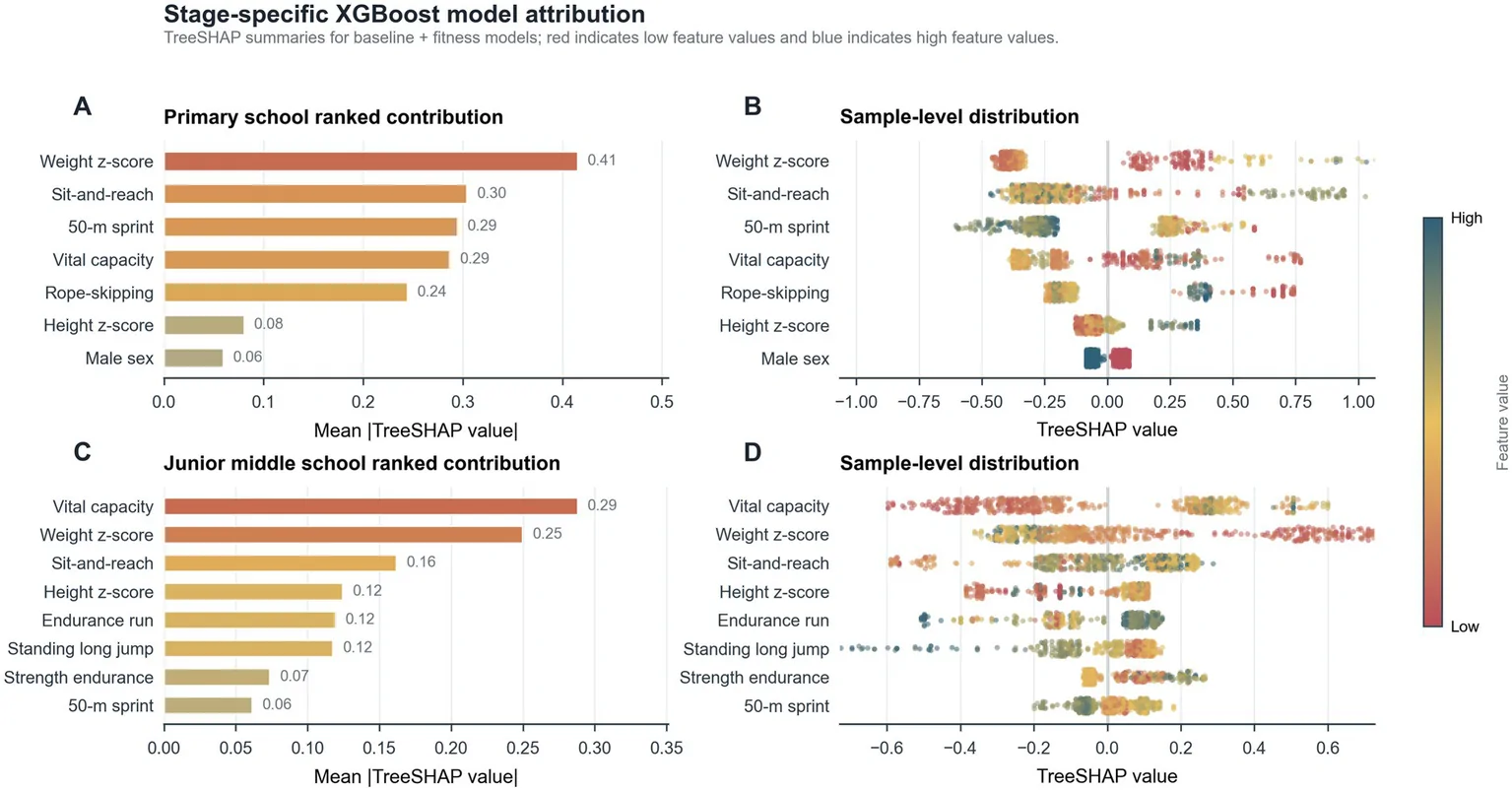
Stage-specific XGBoost model attribution. **(A,C)** Ranked mean absolute TreeSHAP contributions in primary grade 1 and junior middle grade 1. **(B,D)** Sample-level TreeSHAP distributions for the same features. Positive TreeSHAP values indicate that a feature value moved the fitted model toward classification as screening-defined current myopia, whereas negative values moved it away. Red indicates lower observed feature values and blue indicates higher observed feature values. SHAP values are model-specific attribution measures and should not be interpreted as independent associations or causal effects.

The SHAP summaries show how individual features contributed to each fitted model. Body-weight *z*-score, sit-and-reach, 50-m run, vital capacity, and rope skipping were prominent in primary grade 1. In junior middle grade 1, the leading features included vital capacity, body-weight *z*-score, sit-and-reach, height *z*-score, endurance run, and standing long jump. A positive SHAP value shifted the fitted classification toward current myopia; a negative value shifted it away. Interpretation stops there. SHAP values depend on the model, correlations among features, the sampled population, and the covariates available for fitting. They are not independent associations or causal effects. The vital-capacity pattern, for example, could reflect body size, growth, maturation, correlated fitness traits, or residual confounding (Table 4; Figures 3, 4).

**Table 4:** Machine-learning analysis of physical-fitness profiles and current myopia status.

Grade-stage stratum	Model	AUC, mean (SD)	Brier score, mean (SD)
Primary grade 1	Baseline	0.746 (0.181)	0.147 (0.013)
Primary grade 1	Fitness items	0.824 (0.088)	0.117 (0.010)
Primary grade 1	Baseline + fitness	0.793 (0.179)	0.110 (0.008)
Junior middle grade 1	Baseline	0.648 (0.044)	0.229 (0.009)
Junior middle grade 1	Fitness items	0.716 (0.050)	0.216 (0.005)
Junior middle grade 1	Baseline + fitness	0.721 (0.052)	0.213 (0.006)

## Discussion

We found a sharp cross-sectional difference in current myopia between the two school-entry groups. The higher prevalence in junior middle grade 1 accords with the established relation of myopia to age and educational exposure (1, 3). It should not, however, be read as a trajectory: the study compared different students at a single time point. Ocular development, academic demands, near work, screen use, outdoor exposure, and family or school circumstances could all contribute to the gap, but were not measured here. Sex differences were also more visible in the older group. Without direct measures of the relevant developmental and behavioral factors, any explanation remains tentative.

Lower fitness category and lower motor-fitness performance were associated with current myopia, most clearly in primary grade 1. Several explanations remain possible. Physical fitness and myopia may share behavioral, familial, developmental, educational, socioeconomic, or environmental causes; key candidates such as outdoor activity, near work, screen exposure, sleep, academic workload, and parental myopia were absent from the records. Reverse causation is another possibility if children with myopia participate differently in sport or outdoor play. The PRs therefore quantify adjusted cross-sectional associations, not an independent effect of fitness. The primary-grade estimate is especially uncertain because only 43 students in that group met the screening definition.

The XGBoost results add a descriptive view of item-level patterns, but little more. Their AUCs came from internal cross-validation and cannot establish clinical utility. SHAP values indicate where a fitted model placed weight; they do not separate correlated features or recover causal effects. This matters for vital capacity, whose inverse pattern could arise from body size, maturation, correlated fitness characteristics, or unmeasured confounding. Interpretation is further limited by the small number of primary-grade cases and the lack of external validation.

Linking fitness and vision records may still be useful at the population level because it reveals health needs that occur in the same schools. It does not justify selecting individual children for eye examinations on the basis of fitness. Established vision-screening and referral procedures remain essential. Schools may use the two surveillance streams together when planning outdoor opportunities, physical activity, breaks from prolonged near work, screen-use guidance, and visual-health education. Whether such coordination improves outcomes is an empirical question for prospective studies.

The study has important limitations. Its cross-sectional design leaves temporal order unresolved and allows reverse causation. Current myopia was determined by routine non-cycloplegic screening rather than cycloplegic refraction. Residual accommodation can shift refraction in the myopic direction, producing pseudomyopia or false-positive classification, particularly in younger children. The issue may be more consequential in primary grade 1, where mean SE was near emmetropia (+0.08 D), than in junior middle grade 1 (mean SE − 1.76 D). Requiring both unaided visual acuity <5.0 and SE ≤ −0.50 D in the same eye may improve specificity, but it cannot substitute for cycloplegia. We therefore may have overestimated prevalence, especially in the younger group, and paired cycloplegic measurements were unavailable to quantify the bias. Its effect on the fitness-myopia association is uncertain. The ocular-history exclusions removed recorded amblyopia, congenital cataract, corneal disease, retinal disease, and previous ocular surgery; unrecorded or undiagnosed conditions may nonetheless remain in routine data. Important covariates were also missing, including parental myopia, outdoor time, near-work duration, screen exposure, sleep, academic workload, and detailed socioeconomic measures. Residual confounding could explain part, or even all, of the observed associations. Generalizability is narrow because the sample came from three schools in one highly urbanized district. School fixed effects and class-clustered standard errors address some dependence in these data, but three schools do not support reliable school-clustered inference or broad population claims. Fitness results are additionally affected by body size, maturation, sex, effort, and testing conditions. Finally, the machine-learning analysis was exploratory, internally validated only, and requires testing in an external prospective sample.

## Conclusion

Among students from three schools in Xicheng District, failing the official fitness standard and having lower motor fitness were associated with screening-defined current myopia. The association was most evident in primary grade 1, but temporal direction is unknown. Non-cycloplegic misclassification, reverse causation, and unmeasured behavioral, familial, educational, or environmental factors could have shaped the estimates. XGBoost and SHAP described patterns within the study sample; they did not establish a clinically useful prediction tool. Multicenter prospective studies with cycloplegic refraction, stronger confounder measurement, and external validation are needed before any practical screening implication can be considered.

## Data Availability

The raw data supporting the conclusions of this article will be made available by the authors, without undue reservation.
