# Supplementary material for: Association between physical fitness and myopia among school-entry students: a school-based cross-sectional study
Source: Front Public Health. 2026 Sep 3;14:1916100. doi: 10.3389/fpubh.2026.1916100 (PMC13582597; doi:10.3389/fpubh.2026.1916100)
Supplement: Supplementary file 1 [file Table_1.DOCX]

**Attachment 1. Supplementary Table S1**

**Recalculation of the Official Physical-Fitness Total Score According to the National Student Physical Health Standard (2014 Revision)**

*Study title: Association between Physical Fitness and Myopia among School-Entry Students: A School-Based Cross-Sectional Study*

Source standard: National Student Physical Health Standard (2014 revision), Ministry of Education of the People's Republic of China. The table below summarizes the scoring procedure used for the two school-entry grades included in the analysis.

**Panel A. Tested items and official item weights used in the analytic sample**

| Domain | Raw indicator | Unit | Primary grade 1: item / weight | Junior middle grade 1 boys: item / weight | Junior middle grade 1 girls: item / weight |
| --- | --- | --- | --- | --- | --- |
| Body morphology | Body mass index (BMI) | kg/m² | BMI / 15% | BMI / 15% | BMI / 15% |
| Cardiopulmonary function | Vital capacity | mL | Vital capacity / 15% | Vital capacity / 15% | Vital capacity / 15% |
| Speed | 50-m run | s | 50-m run / 20% | 50-m run / 20% | 50-m run / 20% |
| Flexibility | Sit-and-reach | cm | Sit-and-reach / 30% | Sit-and-reach / 10% | Sit-and-reach / 10% |
| Coordination | 1-min rope skipping | counts/min | Rope skipping / 20% | Not applicable | Not applicable |
| Lower-limb power | Standing long jump | cm | Not applicable | Standing long jump / 10% | Standing long jump / 10% |
| Muscular strength/endurance | Pull-ups | counts | Not applicable | Pull-ups / 10% | Not applicable |
| Muscular strength/endurance | 1-min sit-ups | counts/min | Not applicable | Not applicable | 1-min sit-ups / 10% |
| Endurance | Endurance run | min:s | Not applicable | 1000-m run / 20% | 800-m run / 20% |

Notes: The item composition and weights follow the National Student Physical Health Standard (2014 revision). Time-based running items were scored in the direction specified by the official standard; for z-score analyses, lower time was reverse-coded to indicate better performance.

**Panel B. Item-score conversion, bonus-score rules, and final-score calculation**

| Step | Procedure used in this study | Operational detail |
| --- | --- | --- |
| 1. Raw-score checking | Check grade, sex, measurement unit, and plausible value range for each raw physical-fitness indicator. | Records with missing key exposure variables, absence from testing, or implausible values after data checking were excluded from the analytic dataset. |
| 2. Single-item conversion | Convert each raw indicator into an official single-item score using the grade- and sex-specific lookup tables in the national standard. | For performance-based items, a higher single-item score indicates better performance after official conversion. For time-based items, a shorter time corresponds to a better official score. |
| 3. Standard score | Calculate the weighted standard score. | Standard score = Σ(single-item score × official item weight / 100). The maximum standard score is 100. |
| 4. Bonus score | Add official bonus points only for designated high-excellence items. | Primary grade 1: 1-min rope skipping, maximum 20 bonus points. Junior middle grade 1 boys: pull-ups and 1000-m run, maximum 10 points for each item. Junior middle grade 1 girls: 1-min sit-ups and 800-m run, maximum 10 points for each item. Total bonus score is capped at 20 points. |
| 5. Official total score | Calculate the academic-year total score. | Official total score = standard score + bonus score. The maximum total score is 120. |
| 6. Fitness level | Classify the official fitness level according to the national standard. | Excellent: ≥90.0; Good: 80.0–89.9; Pass: 60.0–79.9; Fail: ≤59.9. |

Note: The bonus-score step was applied according to the official high-excellence item rules. If an item did not exceed the official 100-point threshold, no bonus point was assigned for that item.

**Panel C. Derived physical-fitness variables used in statistical and machine-learning analyses**

| Derived variable | Construction | Use in the manuscript |
| --- | --- | --- |
| Official fitness total score | Academic-year total score recalculated from official single-item scores, weights, and bonus-score rules. | Modeled continuously per 10-point lower score and presented in restricted cubic spline analysis. |
| Official fitness level | Excellent, good, pass, and fail categories based on the official total score. | Used as a categorical exposure in modified Poisson regression, with excellent as the reference group. |
| Motor-fitness z-score | Raw motor items were standardized within the relevant grade-stage stratum. Time-based running items were reverse-coded before standardization so that higher z-scores consistently indicated better motor performance. The motor-fitness score was calculated as the mean of available standardized motor items. | Modeled per 1-SD lower score. For primary grade 1, motor items mainly included 50-m run, sit-and-reach, and rope skipping. For junior middle grade 1, motor items included 50-m run, sit-and-reach, standing long jump, sex-specific strength/endurance item, and endurance run. |
| Functional-fitness indicator | Vital capacity was retained as a separate indicator because it is strongly influenced by growth, maturation, body size, and cardiopulmonary function. | Used in domain-specific analyses and machine-learning feature sets. |
| Anthropometric indicators | Height, body weight, and BMI were calculated from routine physical-examination records; height and body weight z-scores were used in machine-learning baseline models. | Used as covariates or baseline features. BMI was adjusted for in regression models. |

*Abbreviations: BMI, body mass index; SD, standard deviation.*

Reference for scoring standard: Ministry of Education of the People's Republic of China. National Student Physical Health Standard (2014 revision). Beijing: Ministry of Education; 2014.
