# Supplementary material for: Association between physical fitness and myopia among school-entry students: a school-based cross-sectional study
Source: Front Public Health. 2026 Sep 3;14:1916100. doi: 10.3389/fpubh.2026.1916100 (PMC13582597; doi:10.3389/fpubh.2026.1916100)
Supplement: Supplementary file 2 [file Table_2.DOCX]

**Supplementary Table S2**

**School-fixed-effect and class-clustered sensitivity analysis**

Association of physical-fitness indicators with screening-defined current myopia after accounting for school and class structure.

| **Indicator** | **n / events** | **Adjusted PR (95% CI)** | **P value** |
| --- | --- | --- | --- |
| Fitness total score, per 10-point lower | 1,296 / 496 | 1.05 (0.97–1.14) | 0.220 |
| Fitness level: Excellent | 429 / 119 | 1.00 (reference) | — |
| Fitness level: Good vs Excellent | 504 / 217 | 1.10 (0.92–1.32) | 0.277 |
| Fitness level: Pass vs Excellent | 327 / 134 | 1.03 (0.87–1.24) | 0.700 |
| Fitness level: Fail vs Excellent | 36 / 26 | 1.95 (1.13–3.38) | 0.018 |
| Motor fitness, per 1-unit lower composite z-score | 1,296 / 496 | 1.14 (0.99–1.31) | 0.078 |
| Primary grade 1: total score, per 10-point lower | 642 / 43 | 1.37 (0.88–2.13) | 0.150 |
| Primary grade 1: motor fitness, per 1-unit lower composite z-score | 642 / 43 | 2.05 (1.13–3.69) | 0.020 |
| Junior middle grade 1: total score, per 10-point lower | 654 / 453 | 1.01 (0.94–1.07) | 0.819 |
| Junior middle grade 1: motor fitness, per 1-unit lower composite z-score | 654 / 453 | 1.02 (0.93–1.13) | 0.615 |

**Note.** PR, prevalence ratio; CI, confidence interval. Modified Poisson regression used a log link. Overall models adjusted for grade stage, sex, BMI, and school fixed effects; grade-stage-specific models adjusted for sex, BMI, and school fixed effects. Standard errors were clustered by class (36 classes overall; 18 within each grade-stage stratum). Confidence intervals and P values used Student t inference with 35 degrees of freedom overall and 17 degrees of freedom in grade-stage-specific models. School-level clustered inference was not used because only three schools were available. Motor fitness was the mean of available item-level z scores standardized within grade stage; running-time items were reverse-coded so that higher values represented better performance.

**Interpretation.** The fail-versus-excellent association and the primary-grade motor-fitness association remained statistically evident after accounting for school and class structure. Other estimates were directionally similar but less precise and should not be described as statistically robust.
